# Impairment of Instrumental Activities of Daily Living in Patients with Mild Cognitive Impairment

**DOI:** 10.4306/pi.2009.6.3.180

**Published:** 2009-08-03

**Authors:** Inn Sook Ahn, Ji-Hae Kim, Seonwoo Kim, Jae Won Chung, Hyeran Kim, Hyo Shin Kang, Doh Kwan Kim

**Affiliations:** 1 Center for Clinical Research, Samsung Biomedical Research Institute and Samsung Medical Center, Sungkyunkwan University School of Medicine, Seoul, Korea.; 2 Department of Psychiatry, Samsung Medical Center, Sungkyunkwan University School of Medicine, Seoul, Korea.; 3 Biostatistics Unit, Samsung Biomedical Research Institute, Seoul, Korea.

**Keywords:** Mild cognitive impairment, Instrumental activities of daily living, Seoul-Instrumental Activities of Daily Living

## Abstract

**Objective:**

This study was conducted to examine the following: whether patients with mild cognitive impairment (MCI) show impairments in instrumental activities of daily living (IADL) as compared to controls; to identify the functional sub-domains of instrumental activities of daily living (IADL) that are affected in MCI and, finally, to identify the Seoul-Instrumental Activities of Daily Living (S-IADL) scale cut-off score that best differentiated between MCI and controls.

**Methods:**

This study was carried out at the geropsychiatry clinic, university hospital. The study participants included 66 patients with MCI and 61 normal elderly. The S-IADL and Seoul-Activities of Daily Living (S-ADL) scales were administered to the main caregivers of all participants in order to assess everyday functioning.

**Results:**

The total S-IADL score was significantly higher in the patients with MCI [mean (SD) score=4.47 (2.06)] than in the controls [mean (SD) score=1.44 (1.65)] (p<0.001). The patients with MCI performed significantly worse on IADLs, such as the ability to use the telephone, prepare meals, take medication, manage belongings, keep appointments, talk about recent events, and perform leisure activities/hobbies (p<0.05). The S-IADL scale discriminated well between patients with MCI and controls (Area Under Curve=87%).

**Conclusion:**

The patients with MCI showed impairments in the ability to perform complex ADL in comparison to healthy controls. IADLs related to memory and frontal/executive functioning were particularly affected in MCI.

## Introduction

The cognitive changes associated with degenerative dementias such as Alzheimer's disease (AD), lead to progressive declines in the patient's ability to perform activities of daily living (ADL).^1^ Mild cognitive impairment (MCI) has been conceptualized as a prodromal state of a neurodegenerative disorder such as AD and an intermediate state between normality and mild dementia.^2^ MCI is distinguished from mild dementia by the absence of global intellectual deterioration and significant deficits in ADL.

However, several recent studies have suggested that patients with MCI can experience subtle changes in the ability to perform instrumental activities of daily living (IADL).^1^,^3^-^8^ Perneczky et al.^3^ found that activities involving memory or complex reasoning were particularly impaired, whereas more basic activities were unimpaired. Farias demonstrated that patients with MCI show significantly more functional impairment in the entire functional domain in comparison to healthy controls, and the magnitude of these changes was greatest for those functional abilities that rely heavily on memory.^1^ In addition, some authors have argued that the explicit inclusion of functional declines should be a part of the diagnostic criteria for MCI.^9^ In 2004, the International Working Group on MCI proposed the inclusion of "preserved basic ADL with some minimal impairment in complex instrumental functions" in the diagnostic criteria of MCI.^10^ However, there is currently no consensus regarding how and which IADLs are affected in MCI.^11^ Furthermore, although numerous studies have examined functional impairments in dementia, very few studies have focused on functional change in MCI.

On the basis of the findings of previous studies, we examined the following: whether patients with MCI show impairments in IADL when compared to healthy controls; to identify which functional abilities of IADLs are affected in patients with MCI; and finally, to identify the Seoul-Instrumental Activities of Daily Living (S-IADL)^12^ scale cut-off score that best differentiated between MCI patients and controls using receiver operating characteristic (ROC) curve analysis.

## Methods

### Subjects

This study was carried out at the Geropsychiatry Clinic, Department of Psychiatry, Samsung Medical Center in Seoul. The study participants included 66 patients with MCI (male=21, female=45) and 61 normal older controls (male=13, female=48). The patients were diagnosed with MCI according to the following research criteria: 1) cognitive decline by self and/or informant reporting; 2) cognitive impairment on objective cognitive tasks - cognitive performance of 1.5 SD below the age and education norm in one or more of the following domains: memory, language, visuoconstruction, and frontal/executive function; 3) essentially preserved activities of daily living; 4) does not meet criteria for dementia. MCI patients were classified as amnestic MCI (aMCI, n=48) if they had a prominent memory impairment either alone or with other cognitive impairments, or as nonamnestic MCI (naMCI, n=18) if a single nonmemory domain was impaired alone or in combination with other nonmemory impairments.

All patients underwent a comprehensive neuropsychological battery, neurologic evaluation, and appropriate laboratory work. A battery of neuropsychological tests was administered to assess the general cognitive functioning and cognitive domains frequently affected by dementia, i.e., verbal and nonverbal memory, language, visuocons-tructive functioning, and frontal related abilities. Table 1 summarizes the neuropsychological measures utilized in the study. Global severity of disease was assessed using the Korean version-Mini Mental State Examination (K-MMSE)^13^ and Clinical Dementia Rating (CDR)^14^ scale. All patients were rated as CDR stage 0.5. Patients with other neurologic or psychiatric disorders or clinically significant medical conditions were excluded. None of the patients had a history of head trauma, seizure, and alcohol or drug abuse.

The control subjects were recruited by an advertisement. They were determined to be cognitively, functionally, and neurologically intact healthy older adults based on the following criteria: 1) free of memory and cognitive disorders; 2) live independently without difficulty; 3) no active psychiatric illness, alcohol/drug history; and 4) free of significant underlying medical and neurological conditions. The control subjects were clinically evaluated based on the Health Screening Exclusion Criteria descried by Christensen et al.^15^ by a psychiatrist to ensure the absence of medical and psychiatric problems. In addition, a psychiatrist administered neurological examination to each subject, and an independent clinical psychologist administered several neuropsychological tests to evaluate cognitive functions. Following the completion of those procedures, controls were characterized as "normal". The protocol was approved by the ethics review board of Samsung Medical Center, Seoul, Korea. Signed informed consent was obtained from all participants.

### Assessment of everyday function

The S-IADL and S-ADL^16^ scales were administered to the main caregivers of all MCI patients and healthy controls by clinical psychologist. The S-IADL was developed to assess patients' abilities to perform instrumental and social activities of daily living. These include the ability to prepare a balanced meal, remember appointments, keep financial records, remember to take medication, and so on. It is composed of 15 items, and the possible score ranges from 0 to 45. The lower scores are indicative of better functioning. The S-ADL was developed to assess the basic activities of daily living, including personal hygiene care, toileting, dressing, bathing, and so on. It is composed of 12 items, and the possible score ranges from 0 to 24. The lower scores are indicative of better functioning.

### Statistical analysis

Multiple logistic regression analyses adjusted for age, sex, and education were used to determine whether the MCI patients showed impairments in IADL when compared to the controls and to determine which domains of IADL were more impaired in MCI. Wilcoxon rank sum test was used to determine whether the patients with MCI showed impairments in basic ADL and IADL when compared to the controls. A ROC curve was used to identify the optimal cut-off scores on the S-IADL, which differentiated best between the patients with MCI and normal controls. The Fisher's exact test and Wilcoxon rank sum test were used to assess the differences in age, sex, education, and K-MMSE score between the patients with MCI and controls. All statistical analyses were performed using SAS software version 9.13 and Statistical Package for Social Science (SPPS; SPSS Inc., Chicago, IL, USA) version 12.0.

## Results

### Demographic characteristics

There were significant differences in age and K-MMSE score between the MCI patients and normal control group (p<0.001)(Table 2). The patients with MCI [mean (SD) age=70.76 (7.33) years] were older than the controls [mean (SD) age=64.44 (5.60) years]. The patients with MCI scored significantly lower on the K-MMSE than the controls. The mean (SD) K-MMSE scores were 24.77 (3.10) in the patients with MCI and 27.64 (1.44) in the controls.

### Differences in activities of daily living performance between the patients with mild cognitive impairment and controls

The total S-IADL score was significantly higher in the patients with MCI [mean (SD) score=4.47 (2.06)] than in the controls [mean (SD) score=1.44 (1.65)] (p<0.001). The results of logistic regression analysis adjusted for age, sex, and education showed that there were significant differences between the two groups on the total S-IADL score (OR=2.07; 95% CI=1.59-2.71; p<0.001) and IADLs, such as the ability to use the telephone (OR=10.55; 95% CI=2.66-41.81; p=0.001), prepare meals (OR=3.33; 95% CI=1.65-6.70; p=0.001), take medication (OR=4.40; 95% CI=1.26-15.35 p=0.020), manage belongings (OR=2.86; 95% CI=1.41-5.78; p=0.004), keep appointments (OR=8.12; 95% CI=2.23-29.62; p=0.002), talk about recent events (OR=2.90; 95% CI=1.41-5.97; p=0.004), and perform leisure activities/hobbies (OR=3.01; 95% CI=1.33-6.81; p=0.008)(Table 3).

There were no significant differences in the S-ADL score between the patients with MCI and controls (p>0.05). All participants were able to perform basic activities of daily living including personal hygiene care, toileting, dressing, and so on, without assistance.

### Accuracy of discriminating two groups

**FIGURE 1:**
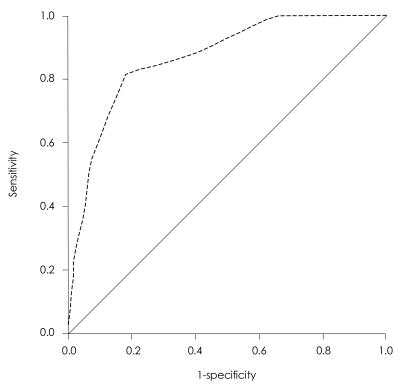
ROC curve for the S-IADL. ROC: receiver operating characteristic, S-IADL: Seoul-Instrumental Activities of Daily Living.

The S-IADL scale discriminated well between patients with MCI and normal controls (Figure 1). When the optimal cut-off value was 3, the sensitivity and specificity were 82% and 82%, respectively. The diagnostic accuracy of the S-IADL was high in the differentiation of the patients with MCI and controls (Area Under Curve=87%).

## Discussion

Our results showed that patients with MCI performed significantly poorer on the IADL than the controls. These results are consistent with previous studies showing that patients with MCI show greater impairment in IADL performance than age- and gendermatched cognitively unimpaired controls.^1^,^3^,^4^ MCI patients have mild cognitive impairment in single or multiple domains, including memory function. In addition, such cognitive impairment may bring about minimal changes in everyday life. Based on our clinical experiences, patients with MCI can independently perform their social activities and usually maintain their occupations. However, subtle changes, such as repeating minor mistakes at work, decreased efficiency, or slow performance, are observed in patients with MCI. In a previous longitudinal study, IADL-restricted MCI subjects were more likely to develop dementia over the course of 2 years than non-restricted MCI subjects. In addition, the restriction of IADL was associated with a much lower chance of reversibility to normal cognition.^17^ In other words, MCI patients with IADL restriction may be more likely to relate to pathologic processes, such as dementia. These findings suggest that the restrictions in IADL in patients with MCI have a strong diagnostic value for subsequent dementia. Further longitudinal studies are still needed to assess the impact of IADL restriction on the progression to dementia and on the reversibility of MCI.

Do patients with MCI show functional deficits in all of the IADLs? Or is there a specific domain that is sensitive to cognitive impairment? Perneczky et al.^3^ suggested that MCI patients have limitations in the ability to perform everyday tasks that involve either memory, such as the ability to keep appointments and find things, or complex reasoning, such as the ability to keep financial records and prepare meals. Farias et al.^1^ showed that patients with MCI were impaired relative to the normal controls on the domains, which require episodic memory for recently acquired information. In our study, IADLs related to memory functioning and frontal/executive functioning were affected in MCI patients. MCI patients showed significantly more impairment in the areas of 'using the telephone', 'preparing meals', 'taking medication', 'managing belongings', 'keeping appointment', 'talking about recent events', and 'leisure/hobbies' than normal elderly controls.

Memory impairment is one of the neuropsychologic domains most frequently affected in MCI. Thus, the domains of everyday functioning that tend to be affected in MCI broadly correspond to the types of impairments that are seen on formal neuropsychological testing. Further, the presence of changes in memory-related activities is consistent with most of the neuroanatomic changes commonly observed in MCI. On the other hand, frontal/executive dysfunction is not generally predominant on formal neuropsychological testing in MCI. However, our findings and those of previous studies suggest that MCI patients already experience functional impairment in the ability to perform everyday activities requiring higher-level executive functioning, such as using the telephone and preparing meals. Those discrepancies indicate that informant-rated measures of actual everyday function may be a more sensitive predictor of disease than objective tests evaluating frontal lobe abilities. In addition, the assessment of complex ADL is very important for diagnosis of MCI.

Finally, our findings suggest that the S-IADL scale, which evaluates complex ADL based on an informant interview, discriminated well between patients with MCI and normal controls. When the optimal cut-off value was 3, the sensitivity and specificity were 82% and 82%, respectively. These results indicate that the S-IADL is a useful instrument for the diagnosis of MCI. In addition, the assessment of functional impairments by caregiver reporting may help differentiate patients with MCI from normal older adults. In the clinical setting, neuropsychological tests are mostly influenced by education and gender of the patients. However, informant-rated measures of everyday function can be less influenced by these types of variables, which may increase the sensitivity in the detection of MCI.

This study has several limitations. The cross-sectional design of this study is one limitation of this study. Extensive longitudinal research is needed in order to clarify our findings. Patients with MCI are known to be a heterogeneous group; therefore, it is possible that different patterns of functional impairment may predominate depending on the subtype of MCI. Further study should be conducted to clarify this issue. Finally, functional decline may be influenced by various variables such as depression or vascular factors. However, this study did not control clearly these effects.

## Figures and Tables

**TABLE 1:**
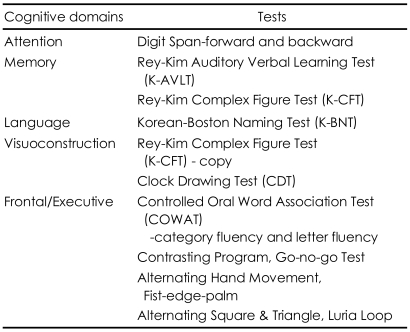
Neuropsychological test measures

**TABLE 2:**
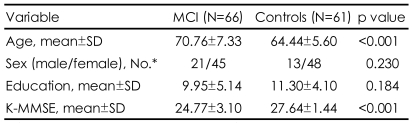
Demographic characteristics of the study population (N=127)

Wilcoxon rank sum test was used. ^*^Fisher's exact test was used. MCI: mild cognitive impairment, K-MMSE: Korean version-Mini Mental State Examination

**TABLE 3:**
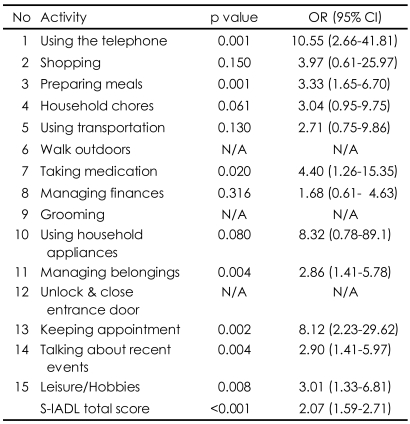
Results of logistic regression analysis (N=127)

Multiple logistic regression analysis adjusted for age, sex, and education was used. Data of the S-IADL 6, 9, 12 was not available for Multiple logistic regression analysis. OR: odds ratio, CI: confidence interval, S-IADL: Seoul-Instrumental Activities of Daily Living

## References

[1] Farias ST, Mungas D, Reed BR, Harvey D, Cahn-Weiner D, DeCarli C (2006). MCI is associated with deficits in everyday functioning. Alzheimer Dis Assoc Disord.

[2] Petersen RC (2004). Mild cognitive impairment as a diagnostic entity. J Intern Med.

[3] Perneczky R, Pohl C, Sorg C, Hartmann J, Tosic N, Grimmer T (2006). Impairment of activities of daily living requiring memory or complex reasoning as part of the MCI syndrome. Int J Geriatr Psychiatry.

[4] Perneczky R, Pohl C, Sorg C, Hartmann J, Komossa K, Alexopoulos P (2006). Complex activities of daily living in mild cognitive impairment: conceptual and diagnostic issues. Age Ageing.

[5] Griffith HR, Belue K, Sicola A, Krzywanski S, Zamrini E, Harrell L (2003). Impaired financial abilities in mild cognitive impairment: a direct assessment approach. Neurology.

[6] Nygård L (2003). Instrumental activities of daily living: a stepping-stone towards Alzheimer's disease diagnosis in subjects with mild cognitive impairment?. Acta Neurol Scand Suppl.

[7] Tabert MH, Albert SM, Borukhova-Milov L, Camacho Y, Pelton G, Liu X (2002). Functional deficits in patients with mild cognitive impairment: prediction of AD. Neurology.

[8] Artero S, Touchon J, Ritchie K (2001). Disability and mild cognitive impairment: a longitudinal population-based study. Int J Geriatr Psychiatry.

[9] Ritchie K, Artero S, Touchon J (2001). Classification criteria for mild cognitive impairment: a population-based validation study. Neurology.

[10] Winblad B, Palmer K, Kivipelto M, Jelic V, Fratiglioni L, Wahlund LO (2004). Mild cognitive impairment-beyond controversies, towards a consensus: report of the International Working Group on Mild Cognitive Impairment. J Intern Med.

[11] Okonkwo OC, Wadley VG, Griffith HR, Ball K, Marson DC (2006). Cognitive correlates of financial abilities in mild cognitive impairment. J Am Geriatr Soc.

[12] Ku HM, Kim JH, Kwon EJ, Kim SH, Lee HS, Ko HJ (2004). A Study on the reliability and validity of Seoul-Instrumental Activities of Daily Living (S-IADL). J Korean Neuropsychiatr Assoc.

[13] Kang Y, Na DL, Hahn S (1997). A validity study on the Korean Mini-Mental State Examination (K-MMSE) in dementia patients. J Korean Neurol Assoc.

[14] Morris JC (1993). The Clinical Dementia Rating (CDR): Current version and scoring rules. Neurology.

[15] Christensen KJ, Multhaup KS, Nordstrom S, Voss K (1991). A Cognitive Battery for Dementia: Development and Measurement Characteristics. Psychol Assesst.

[16] Ku HM, Kim JH, Lee HS, Ko HJ, Kwon EJ, Jo S (2004). A Study on the reliability and validity of Seoul-Activities of Daily Living (S-ADL). J Korean Geriatr Soc.

[17] Pérès K, Chrysostome V, Fabrigoule C, Orgogozo JM, Dartigues JF, Barberger-Gateau P (2006). Restriction in complex activities of daily living in MCI: Impact on outcome. Neurology.

